# Social Media and Wellness: Evolving Trends in Orthopaedic Surgery Residency Representation

**DOI:** 10.5435/JAAOSGlobal-D-25-00321

**Published:** 2025-11-10

**Authors:** Abdullah Chandasir, Krishna N. Chopra, Nicole L. Greene, Anna L. Gorsky, Jesse Seilern und Aspang, Sameer R. Khawaja, Michael B. Gottschalk, Amanda L. Dempsey, Roberto C. Hernandez-Irizarry, Eric R. Wagner

**Affiliations:** From the Medical College of Georgia, Augusta, GA (Chandasir); the Department of Orthopaedic Surgery, Emory University School of Medicine, Atlanta, GA (Dr. Chopra, Greene, Gorsky, Dr. Seilern und Aspang, Dr. Gottschalk, Dr. Dempsey, Dr. Hernandez-Irizarry, and Dr. Wagner); and the Department of Orthopaedic Surgery, Baylor College of Medicine, Houston, TX (Dr. Khawaja).

**Keywords:** residency, social media, wellness, online engagement, burnout, orthopaedic surgery

## Abstract

**Introduction:**

Social media is increasingly popular among orthopaedic surgery residency programs and allows for programs to communicate with current and prospective residents. One way is by showcasing health and wellness initiatives—a critical response to the burnout many orthopaedic surgery residents face. This study investigates trends in wellness-related social media activity among US orthopaedic surgery residency programs to understand the effect wellness initiatives and their representation on social media have on online engagement and program ranking.

**Methods:**

The Orthopaedic Residency Information Network was queried for program information including location, total number of residents, and whether the program has diversity initiatives. Instagram and Twitter/X were used to identify categorized wellness posts between July 1, 2023, and June 30, 2024. Engagement rates and a summated wellness score were then calculated.

**Results:**

A total of 171 orthopaedic surgery residency programs were reviewed. Instagram was the preferred platform for programs, with resident meet-and-greet/mentorship posts being the most common. Programs with higher Doximity rankings, larger resident cohorts, and diversity initiatives posted more wellness-related content. Greater summated wellness scores were also associated with an increased Instagram follower count. Regional differences were also noted, with programs in the Midwest emphasizing greater work-life balance.

**Conclusion:**

This study highlights the association between orthopaedic surgery residency programs leveraging social media to promote a culture of wellness and improved program perception and rankings, which may play a role in attracting top applicants. Residency-related wellness content remains limited, suggesting opportunities for programs to enhance engagement and resident support through strategic social media efforts.

Social media has become increasingly popular over the past decade, particularly in the medical field. Specialties ranging from dermatology to orthopaedic surgery have increasingly leveraged social media for education and outreach and fostering engagement with patients and colleagues alike. Studies have shown that more than half of all orthopaedic surgeons across multiple subspecialties have at least one professional social media account, with LinkedIn, Instagram, and Twitter/X being the most popular.^1,2^ Growing social media utilization amongst orthopaedic surgeons has also been positively associated with patient satisfaction and research productivity.^3-6^ This trend is also reflected among orthopaedic surgery residency programs, which demonstrated a 300% increase in social media use in 2019.^7^ This increase in social media usage coincided with the COVID-19 pandemic, with 60% of all orthopaedic surgery residency program accounts being created after the onset of COVID to engage with prospective residents.^8,9^ Residency programs use social media in a variety of ways to provide program information and communicate with current and prospective residents. Social media has also proven to be an effective tool in orthopaedic surgery residency recruitment.^10^ Particularly when used with a holistic approach that includes content beyond the clinical setting,^11^ social media has demonstrated to be an important resource for prospective applicants and their perceptions of residency programs.^12^ In addition, programs with a substantial social media presence were found to have increased sex and racial diversity in applicants as well as an increased application volume.^13^

Regarding social media content, residency programs can engage with a range of topics, including the promotion of resident life and well-being initiatives. In the United States, physicians are twice as likely to be affected by burnout than members of the average population.^14^ Within orthopaedic surgery, 52% of residents report feeling burnt out, and 13% report screening positive for depression.^15^ It has become increasingly important for residency programs to promote and cultivate a positive culture among their cohorts, not only to build camaraderie and combat burnout among current residents but also to attract future residents. There is a scarcity of literature on the associations between social media presence, content, and strategy in orthopaedic surgery residency programs and various program metrics, such as rankings and reputation. Although social media may serve as a valuable tool for recruitment and program visibility, its correlation with resident wellness remains uncertain. This study investigates wellness-related social media activity among orthopaedic surgery residency programs across multiple platforms to identify which initiatives performed by programs are most prevalent. In addition, this study aims to identify trends regarding program size, diversity, region, and ranking in relation to wellness-related activity on social media.

## Methods

Descriptive and categorical information on orthopaedic surgery residencies was collected through the American Orthopaedic Association Orthopaedic Residency Information Network. Information collected included name, region, number of residents, and participation in diversity initiatives. Participation in diversity initiatives was recorded as yes/no. For standardization, we did not include information about what specific diversity initiatives programs employed, given that several programs did not expand on these initiatives in their Orthopaedic Residency Information Network profiles. Program reputation ranking was collected from the Doximity Residency Navigator. Rankings are determined by surveying board-certified physicians and asking them to rank residency programs in their respective fields. Alumni status, program size, graduation year, and program director input all influence reputation ranking.^16^

Residency programs were then searched on Instagram and Twitter/X for posts from July 1, 2023, to June 30, 2024. The number of followers, total number of posts related to wellness, and types of wellness-related posts were recorded. Categories of wellness-related posts—determined based on previously published literature^17^—included resident work-life balance, team-building activities, attendance to physical health, fostering a healthy work environment, activities/lectures promoting wellness, and meet and greet/mentorship posts. Posts were assigned to a single classification based on the category to which they were most closely related.

The engagement rate of each social media page was calculated using the Phalanx Instagram and Twitter/X Engagement Rate Calculator. Engagement refers to how a page's audience interacts with posts, including likes, comments, or shares on a post or tweet. Engagement rate is calculated as the total number of interactions (likes, comments, shares) divided by the number of followers and then multiplied by 100 to get a percentage.^18^ A summated wellness score was then calculated to rank how many wellness-related categories each program posted on Instagram and Twitter/X. Statistical analysis was performed on R studio v06.1 and included the Wilcoxon Rank-Sum, Spearman correlation, and analysis of variance tests.

## Results

A total of 171 residency programs were included in this review. Our analysis demonstrated that programs tended to post more wellness content on Instagram and also were more active overall on Instagram than Twitter/X. Of all programs analyzed, 132 (77.2%) programs had active Instagram pages, whereas only 28 (16.4%) programs had active Twitter/X accounts. On Instagram, the mean number of followers was 1,866, with an average engagement rate of 6.6. On Twitter/X, the mean number of followers was 870, with an average engagement rate of 1.4 (Table 1).

**Table 1:** Descriptive Statistics of Orthopaedic Surgery Residency Social Media

Factor or Variable	Number (n = 171)
Active social media accounts	
Instagram	132 (77.2%)
Twitter/X	28 (22.2%)
No. of followers	
Instagram	1865.9 ± 1149.8
Twitter/X	870.4 ± 892.2
Engagement rate	
Instagram	6.6 ± 3.4
Twitter/X	1.4 ± 2.9
Wellness posts in a year	
Instagram	10.4 ± 6.9
Twitter/X	2.4 ± 3.4
Region	
Northeast	49 (28.7%)
Midwest	43 (25.1%)
Southeast	37 (21.6%)
West	24 (14.0%)
Southwest	18 (10.5%)

Values are reported as number (%) or mean ± standard deviation.

The average number of wellness posts on Instagram was 10.4, with the most common category being “meet/greet the resident or mentorship” posts. Of the 132 programs with Instagram, 129 (97.7%) pages had at least one post in this category, with the average number of “meet/greet the resident or mentorship” posts being 17.9, when present (Table 2). On Twitter/X, the average number of wellness-related posts was 2.4, with the most common category also being “meet/greet the resident or mentorship” posts. When present, the average number of tweets in this category was 4.2, with 20 (71.4%) of 28 residency programs with Twitter/X accounts posting such content.

**Table 2:** Wellness Post Category Representation on Instagram

Wellness Category	No. of Residency Programs	Average No. of Posts Per Program
Resident work-life balance	61 (46.2%)	2.3 ± 1.8
Team-building activities	104 (78.8%)	3.2 ± 2.7
Attendance to physical health	22 (16.7%)	0.9 ± 0.9
Healthy work environment	15 (11.3%)	0.9 ± 1.3
Activities/lectures promoting wellness	14 (10.6%)	0.6 ± 0.6
Meet/greet the residents	129 (97.7%)	17.9 ± 133.0

There were not enough active Twitter/X accounts to appropriately report this information.

Values are reported as number (overall percentage) of active Instagram accounts (n = 132) or mean ± standard deviation.

**Figure 1:**
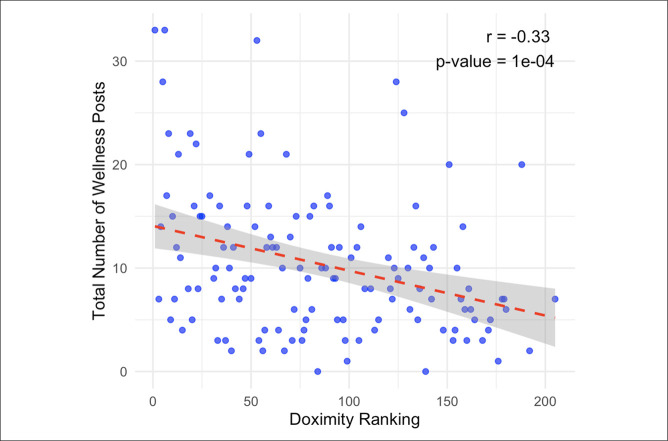
Graph showing relationship between Doximity rank and total number of wellness posts.

Trends in wellness-related posts were associated with different characteristics such as program size and ranking. The number of wellness posts on Instagram was positively correlated with Doximity reputation ranking (*P* < 0.001, *r* = −0.33; Figure 1) and the number of residents in the program (*P* < 0.001, *r* = 0.24). Programs that participated in diversity initiatives had a greater number of wellness posts (12.0 ± 6.9) on average than programs that did not participate in diversity initiatives (8.4 ± 6.4; *P* < 0.001; Table 3). Given the limited number of programs on Twitter/X, there was no correlation on the platform between the number of wellness-related posts or any residency program characteristic (Table 4).

**Table 3:** Program Characteristics and Number of Wellness Posts on Instagram

Category		Mean Number of Wellness Posts	*P* Value
Region			
	Northeast	9.6 ± 6.9	0.410
	Midwest	11.7 ± 6.6	
	Southeast	10.3 ± 7.7	
	West	11.8 ± 7.4	
	Southwest	7.8 ± 4.3	
Participation in diversity initiatives	Yes	12.0 ± 6.9	**0.003**
	No	8.4 ± 6.4	

Category	Correlation (r) for Mean Number of Wellness Posts	*P* Value	
No. of residents	0.33	**<0.001**	
Doximity ranking	0.33	**<0.001**	
No. of followers	0.15	0.090	
Engagement rate	−0.07	0.430	

Bold *P* value denotes statistical significance. Values are reported as mean ± standard deviation.

**Table 4:** Program Characteristics and Number of Wellness Posts on Twitter/X

Category		Mean Number of Wellness Posts	*P* Value
Region	Northeast	1.8 ± 2.4	0.820
	Midwest	1.9 ± 3.6	
	Southeast	3.6 ± 4.4	
	West	3.3 ± 4.0	
	Southwest	2.1 ± 3.7	
Participation in diversity initiatives	Yes	2.7 ± 3.7	0.490
	No	1.9 ± 3.1	

Category	Correlation (r) for Mean Number of Wellness Posts	*P* Value	
No. of residents	−0.03	0.830	
Doximity ranking	0.15	0.370	
No. of followers	0.13	0.440	
Engagement rate	0.09	0.570	

Bold *P* value denotes statistical significance. Values are reported as mean ± standard deviation.

**Figure 2:**
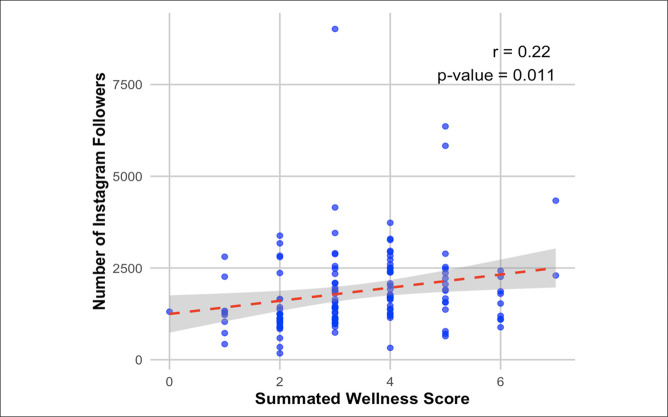
Graph showing relationship between summated wellness score and number of followers.

The average summated wellness score, calculated by awarding one point for each content category represented in posts across Instagram and Twitter/X, was 2.7 ± 1.8 (range: 0 to 7). A higher summated wellness score was positively correlated with program size (*P* < 0.001, *r* = 0.32), Doximity ranking (*P* < 0.001, *r* = 0.43), participation in diversity initiatives (*P* < 0.001), and number of followers (*P* = 0.004; Table 5 and Figure 2). No notable differences were observed in the types of wellness posts by region and Doximity ranking. Residency programs in the Midwest had higher numbers of work-life balance posts on average compared with the rest of the country (*P* = 0.005). Programs with higher Doximity rankings were also more likely to post about team-building activities (*P* = 0.020). Finally, programs that posted about activities/lectures promoting wellness were correlated with increased follower counts (*P* = 0.030).

**Table 5:** Program Characteristics and Summated Wellness Score

Category		Summated Wellness Score	*P* Value
Region	Northeast	2.7 ± 1.6	0.190
	Midwest	3.1 ± 2.1	
	Southeast	2.3 ± 1.6	
	West	3.1 ± 1.9	
	Southwest	2.2 ± 1.7	
Participation in diversity initiatives	Yes	1.7 ± 1.5	**<0.001**
	No	3.6 ± 1.5	

Category	Correlation (*r*) for Mean Number of Wellness Posts	*P* Value	
No. of residents	0.30	**< 0.001**	
Doximity ranking	0.39	**<0.001**	
No. of followers	0.22	**0.010**	
Engagement rate	−0.21	0.070	

Bold *P* value denotes statistical significance. Values are reported as mean ± standard deviation.

## Discussion

Burnout is a pervasive issue in orthopaedic surgery, with more than half of residents reporting symptoms and a notable proportion screening positive for depression.^15^ As residency programs seek to mitigate burnout and foster a culture of wellness, social media has emerged as a dynamic tool for engaging both current and prospective residents.^8,9^ Although previous research has explored social media's role in recruitment, diversity, and education,^13^ its function in promoting and representing wellness initiatives remains inadequately studied. This study aimed to quantify wellness-related social media activity among orthopaedic surgery residency programs in the United States and examine its associations with program size, Doximity ranking, and participation in diversity initiatives.

The findings indicate that despite its potential benefits, wellness-related content remains underutilized by orthopaedic surgery residency programs across social media. Instagram was identified as the predominant platform for engagement, with a markedly higher volume of wellness-related posts compared with Twitter/X. Programs with higher Doximity rankings, larger resident cohorts, and established diversity initiatives demonstrated a greater commitment to wellness-related content. Furthermore, a higher summated wellness score was associated with increased Instagram follower counts, suggesting that a robust social media presence centered on wellness may enhance visibility and engagement. These findings underscore the growing role of social media as not only a communication platform but also a strategic asset for residency programs in shaping public perception, fostering engagement, and attracting high-caliber applicants. One distinct way residency programs can leverage social media is by using it to communicate and reinforce their core values. Beyond simply disseminating information, social media can serve as a powerful medium for demonstrating how a program values and exemplifies key professional attributes, including surgical competence, teaching philosophy, and physician wellness. By highlighting values such as wellness, mentorship, and collaboration, programs can subtly or explicitly display the behaviors, attitudes, and lifestyles they hope to instill in their trainees. In doing so, social media becomes not just a recruitment tool but a formative educational space that influences how residents envision and ultimately live out their roles as attending physicians.

Previous studies across various medical specialties, including neurosurgery and anesthesiology, have emphasized the benefits of structured wellness programs, linking them to reduced burnout and improved resident well-being.^19,20^ The current analysis expands on this literature by addressing a key gap: the extent to which orthopaedic surgery residency programs use social media to highlight and promote wellness initiatives. Comparative analyses with other medical disciplines reveal notable disparities in social media utilization for wellness advocacy. A parallel study among plastic surgery residency programs, for example, reported an average wellness-related post volume more than double that observed among orthopaedic surgery programs.^17^ Similarly, research on ophthalmology residency programs found that applicants highly valued social media content related to resident life,^11^ reinforcing the idea that showcasing wellness initiatives can positively influence resident recruitment.^13^

Correlations between larger program size and increased wellness-related content also align with findings from studies in OB/GYN^21^ and otolaryngology,^22^ where greater social media activity was observed among larger programs. This trend is likely attributable to greater institutional resources, dedicated personnel, and funding for social media management. A regional trend was also observed, with residency programs in the Midwest displaying a heightened focus on work-life balance in their social media content. This variation may be attributed to differences in institutional culture, geographic location, or resource allocation.

This study contributes to the expanding body of literature on residency program engagement strategies, particularly in the realm of wellness promotion. Unlike previous research that primarily focused on social media's role in resident recruitment,^8,9^ these findings highlight the growing importance of wellness as an integral aspect of residency culture. The positive correlation between Doximity ranking and wellness-related social media activity suggests that a program's commitment to residents' well-being may enhance overall satisfaction, ultimately influencing its reputation and desirability among applicants. In addition, a commitment to increased social media activity, particularly in promoting wellness and diversity initiatives, can have a positive effect on increased sex and racial diversity among residency programs.^13^ Seeing images of attending surgeons and current residents that are representative of a variety of races and sexes can offer insight into how programs foster an inclusive learning environment. Prospective residents subsequently may not only feel more confident in applying to these programs that promote diversity and inclusivity but also feel more equally represented among their mentors and peers.^23^ These insights provide a framework for residency programs to optimize their social media strategies, leveraging wellness-related content to enhance recruitment efforts, foster inclusivity, and support resident well-being.

Despite the valuable insights gained from this study, we must acknowledge several limitations. First, the classification of wellness-related posts was necessarily reductive, as certain posts could reasonably belong to multiple categories. This methodological constraint may have led to an underrepresentation of wellness initiatives across programs. Second, the analysis was confined to a specific timeframe, without accounting for fluctuations in engagement over an extended period. The dynamic nature of social media necessitates ongoing assessment to capture evolving trends. In addition, this study did not evaluate the quality or substantive impact of wellness initiatives beyond their representation on social media nor did it consider resident turnover as a potential measure of program well-being. Program size may also serve as a confounding factor, as larger institutions often have greater resources to implement wellness initiatives and maintain a dedicated social media presence. Furthermore, the management of residency social media accounts (whether by faculty, residents, or external administrators) was not assessed, which may influence content strategies and engagement. Residents who may be experiencing burnout themselves may not have the time or energy to keep up with social media accounts, which can lead to a lag in posting content. Finally, this study focused exclusively on Instagram and Twitter/X, potentially overlooking other platforms that are used for communication directly with residents rather than with the general public, as well as overlooking emerging platforms that may hold increasing relevance for applicants and residency programs alike.

## Conclusion

Orthopaedic surgery residency programs demonstrate a preference for Instagram to share wellness-related content, although overall activity remained limited compared with other specialties. Programs with greater engagement in wellness-related posts were correlated with higher Doximity rankings and larger followings, suggesting an association with program visibility and perception. Strengthening social media presence—particularly on Instagram—by highlighting resident life, mentorship, and wellness initiatives may enhance recruitment and engagement. We recognize that social media activity is not a perfect substitute in gauging wellness metrics within orthopaedic surgery residency programs, but we do think there are several ways in which others can expand on our findings. Future research should explore the longitudinal impact of wellness-related social media content on resident well-being, program satisfaction, and recruitment outcomes. Investigating the quality and effectiveness of both wellness and diversity initiatives, beyond their online representation, may provide deeper insights into best practices for resident support and how wellness and diversity influence resident perception of and satisfaction with their program. Finally, examining resident perspectives on social media-driven wellness initiatives may further elucidate their role in fostering a positive training environment, as well as how such initiatives affected residents' perceptions of their own programs.
